# Inferring composition and function of the human gut microbiome in time and space: A review of genome-scale metabolic modelling tools

**DOI:** 10.1016/j.csbj.2020.11.035

**Published:** 2020-12-01

**Authors:** Álvaro Altamirano, Pedro A. Saa, Daniel Garrido

**Affiliations:** Department of Chemical and Bioprocess Engineering, School of Engineering, Pontificia Universidad Católica de Chile, Vicuña Mackenna, 4860 Santiago, Chile

## Abstract

The human gut hosts a complex community of microorganisms that directly influences gastrointestinal physiology, playing a central role in human health. Because of its importance, the metabolic interplay between the gut microbiome and host metabolism has gained special interest. While there has been great progress in the field driven by metagenomics and experimental studies, the mechanisms underpinning microbial composition and interactions in the microbiome remain poorly understood. Genome-scale metabolic models are mathematical structures capable of describing the metabolic potential of microbial cells. They are thus suitable tools for probing the metabolic properties of microbial communities. In this review, we discuss the most recent and relevant genome-scale metabolic modelling tools for inferring the composition, interactions, and ultimately, biological function of the constituent species of a microbial community with special emphasis in the gut microbiota. Particular attention is given to constraint-based metabolic modelling methods as well as hybrid agent-based methods for capturing the interactions and behavior of the community in time and space. Finally, we discuss the challenges hindering comprehensive modelling of complex microbial communities and its application for the *in-silico* design of microbial consortia with therapeutic functions.

## Highlights

• Composition and function are critical for understanding the human gut microbiota.
• Metabolic interplay between host and microbiome is captured by metabolic models.
• A revision of recent metabolic modelling tools and applications is provided.
• Hybrid tools capable of modelling communities in time and space are highlighted.
• Challenges for in silico rational design of microbial consortia are discussed.

## 1 Introduction

Microbiomes could be understood as the collection of microorganisms, their genomes and their surrounding habitat [1]. The human gut hosts a complex and dynamic community of microorganisms that directly modulates gastrointestinal physiology, also playing important roles beyond the digestive system [2]. The gut microbiome is characterized by a large diversity of microbial species belonging to the phyla Firmicutes and Bacteroidetes [3]. Constituent species of these phyla are responsible for the fermentation of dietary substrates into important metabolites including Short Chain Fatty Acids (SCFAs) [4], which serve as energy for the epithelium and modulate important physiological responses [2]. Although gut microbiome profiles are remarkably unique and stable in time for each individual, there are clear transitions in its composition associated with age, diet, antibiotics, or diseases that result in specific signatures [3].

Microbial communities can be studied by the diversity and abundance of their constitutive species, as well as the interactions between them and their environment, e.g., the human host. For instance, composition changes of the intestinal microbiota drive ecological interactions among microbial populations and the host [5], which in the face of external factors or interventions such as diet, drugs, or antibiotics, defines the biological function of the community and its impact on human health [6]. Thus, metabolic interactions between gut microbes and with their host are essential yet they remain largely unknown. Mathematical models of these interactions could help unravel the impact on the microbiome composition upon external interventions, which can be then employed to design microbiome consortia with desired therapeutic functions [7], [8].

Experimental elucidation of microbial interactions is challenging as the number of possible relations grows exponentially with the number of species in the community, thereby hindering implementation of conventional experimental designs. On the other hand, computational methods can readily simulate and propose plausible biological interactions within the gut community, which can be later verified experimentally [9]. Depending on the type of computational framework employed, different aspects of the community can be probed. There are four general approaches used for studying microbial communities [10]: modelling accounting for phenotypic traits [11], [12], [13], modelling based on sequence abundance [14], [15], [16], agent-based modelling [17], [18], [19], and constraint-based methods (CBMs) using metabolic reconstructions from annotated genomes [19], [20], [21], [22]. The first category, usually applied to small communities, employs non-structured Ordinary Differential Equations (ODEs) models to describe the time-course profile of biomass and metabolite concentration changes in the community, resulting from growth activation/inhibition type interactions. The second group applies statistical methods to infer patterns of co-occurrence and co-exclusion, or to estimate physiological traits (e.g., growth rate) based on metagenomic analyses. Agent-based modelling describes different elements of an autonomous system (e.g., populations, metabolites, or environment), interacting with each other and reacting to perturbations based on defined rules. The last group employs genome-scale stoichiometric models and optimization methods to determine the feasible phenotypic space for a metabolic network, or networks in the case of a community. In particular, CBMs for metabolic modelling have emerged as a popular framework for mechanistically describing the behavior of microbial communities in different contexts and for enabling systematic integration of disparate omics data [10], [19], [23]. Furthermore, the continuous development of novel methods and tools has prompted CBMs as the framework of choice for studying microbial communities.

In this review, we review the most relevant metabolic modelling tools for inferring the composition, interactions, and ultimately, the biological function of the constituent species in a microbial community with particular emphasis on the human gut microbiota. Following a brief overview of relevant CBM methods for community modelling, we present recent tools and applications for describing microbial community behavior in time and space, with emphasis but not limited to the human gut. Both time and space dependencies are critical for shaping the collective behavior of the community [22], [24], [25], yet it has not been until recently that more advanced modelling tools have become available for its use and review. Finally, we discuss the challenges of modelling complex microbial communities such as the gut microbiota in time and space, and the steps required for rationally designing effective microbial consortia for tailored applications.

## 2 From genome-scale network reconstructions to stoichiometric models of metabolism

### 2.1 Genome-scale network reconstructions for microbial community modelling

**Fig. 1:**
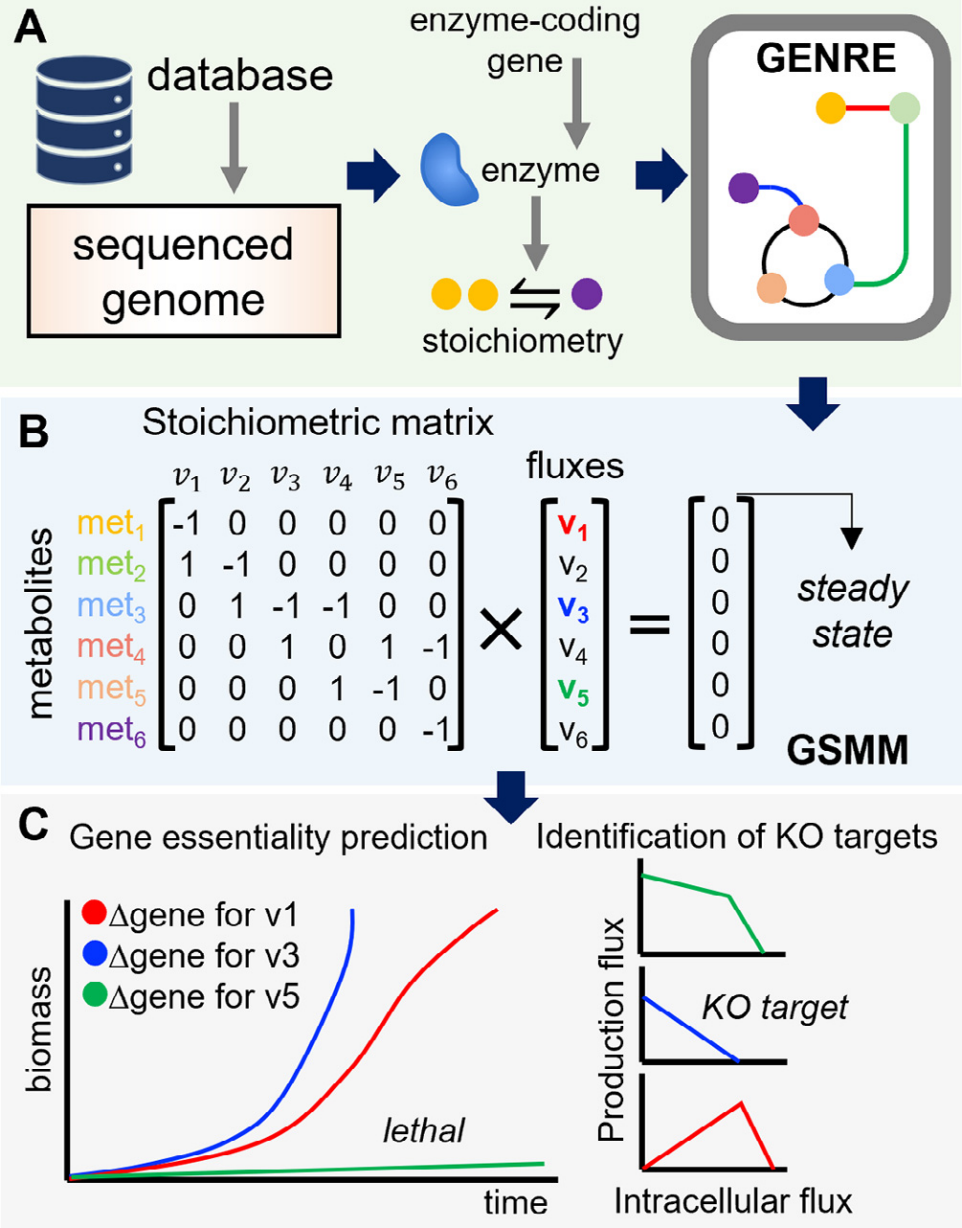
Illustrative applications and construction of GSMM from GENREs. **A** From an annotated genome, Gene-Protein-Reactions (GPR) relations are defined which link genes, enzymes and reactions. The set of all GPRs defines the metabolic network reconstruction. **B** A GSMM is then constructed using the stoichiometric matrix encoding all the reactions stoichiometries and imposing the steady-state condition among other suitable constraints. **C** The GSMM can be then employed for predicting the effect of gene knockouts on growth or simulating the impact of individual intracellular fluxes on the production of a given metabolite, among other uses.

Genome-Scale Metabolic Models (GSMMs) are mathematical structures derived from GEnome-scale Network REconstructions (GENREs; Fig. 1A). The latter represent the complete repertoire of biochemical reactions of a single cell or a microbial community. The collection of reactions, as well as the participating metabolites, are assembled based on the list of enzymes contained in the annotated genome, providing Gene-Protein-Reaction (GPR) relationships that capture the genotype-phenotype relation. Several tools are available for the semi-automatic reconstruction of these networks. Among the most popular are modelSEED [26] and RAVEN [27], among many others [28], which determine the relevant GPRs from the annotated genomic sequence using different databases yielding a draft reconstruction. This draft reconstruction will often require laborious curation, e.g., gap-filling [29], [30]. More recently, a different bottom-up approach called CarveMe [31] has been proposed. Starting from a curated universal model containing reactions from well-curated GSMM databases (e.g., BiGG [32]), CarveMe optimizes the reactions to be maintained in the new reconstruction based on sequence similarity with the annotated genome. Notably, this tool has been used to build high-quality metabolic single-species and community models of the human gut microbiota [31].

The availability of genome sequencing data supported by (semi)automatic tools for GENREs construction has led to the emergence of various network reconstruction resources for exploring the behavior of microbial communities. The most comprehensive resource is AGORA, which is an open repository of over 800 semi-automatically reconstructed GENREs of microorganisms from the human gut [33]. This resource has served as a base for multiple studies, including bile acids metabolism in the human gut [34] and the development of a large human gut microbiota metabolic model that integrates metagenomic data [35]. Another key resource is NJC19 [36], which so far represents the largest literature-based network resource that comprises human and mouse microbiome knowledge. NJC19 serves as a reference map and consists of two groups of nodes; the first group is made up of the considered species (883 microbial species and 6 mouse and human cell types), and the second group contains the metabolites related to each organism. Associations between consumed or produced metabolites by species were either identified from empirical data or predicted by bioinformatic algorithms. Importantly, NJC19 has evidenced a limited number of metabolic phenotypes currently supported as measured by the low average number of metabolites produced or consumed [37]. This information can be very valuable for building and curating metabolic networks.

### 2.2 Genome-scale metabolic models and constrained-based methods

From a defined GENRE, a GSMM can be constructed by mathematically encoding the metabolic reactions in the stoichiometric matrix, which represents the mass balances for each intracellular metabolite located inside the cell. Then, the imposition of the steady-state assumption for the latter enables writing a system of linear equations that represents the metabolic state of the system, here the metabolic network (Fig. 1B). Although the resulting system of equations is typically undetermined, i.e., there are infinite solutions describing the same observations, it is possible to compute particular solutions using CBMs. Briefly, by defining an objective function and applying capacity constraints on the fluxes that simulate specific environmental conditions, the optimal metabolic flux distribution that achieves that goal can be computed. The most popular of such methods is Flux Balance Analysis (FBA) [38], which has been extensively applied for better understanding microbial physiology [39], [40], [41], and more lately, to model microbial communities [42], [43], [44]. Application of FBA in GSMMs are vast and range from gene essentiality predictions to identification of genetic targets for metabolic engineering (Fig. 1C). The reader is referred elsewhere for a more comprehensive review [28].

There are four types of CBMs that have been employed for modelling microbial communities [45]: 1) lumped approach, 2) compartment per guild [35], [46], 3) bi-level optimization simulation [21], [47], and 4) dynamic stoichiometric modelling [48], [49]. The lumped approach models the union of all reactions and metabolites from all the species of the community as if they were one organism with all common and unique metabolic pathways. In the compartment per guild approach, each microorganism is represented as a different compartment, each with its own set of reactions and metabolites. In both cases, popular CBMs such as FBA can be employed to perform metabolic flux predictions. In contrast, bi-level optimization frameworks individualize each microorganism but apply two consecutive rounds of optimization: one for each individual species, and a second for the entire community integrating the results from the previous round. Finally, dynamic stoichiometric modelling methods employ dynamic models coupled to GSMMs to describe dynamic changes in metabolite and biomass concentration across time. The most important of such methods is dynamic FBA (d-FBA) [50]. Briefly, d-FBA employs GSMMs equipped with kinetic expressions (see [51] for more details) for describing the rate of consumption/production of external metabolites consistent with intracellular fluxes. By dynamically updating the latter rates using extracellular concentrations and using them as constraints for FBA, metabolic fluxes and biomass production can be calculated in a specific time. The latter fluxes can be then employed to update the concentrations of the external species. By repeating this procedure iteratively, one can solve the ODE system describing the concentration of biomass and external metabolites as a function of time. Importantly, this method can be readily adapted to model space and temporal dependencies by discretizing the modelled domain and solving the Partial Differential Equations (PDEs) that describe the diffusion of species. The methods reviewed hereafter use extensively this or similar approaches for modeling communities in time and space.

## 3 Metabolic properties of microbial communities inferred from genome-scale metabolic models

**Fig. 2:**
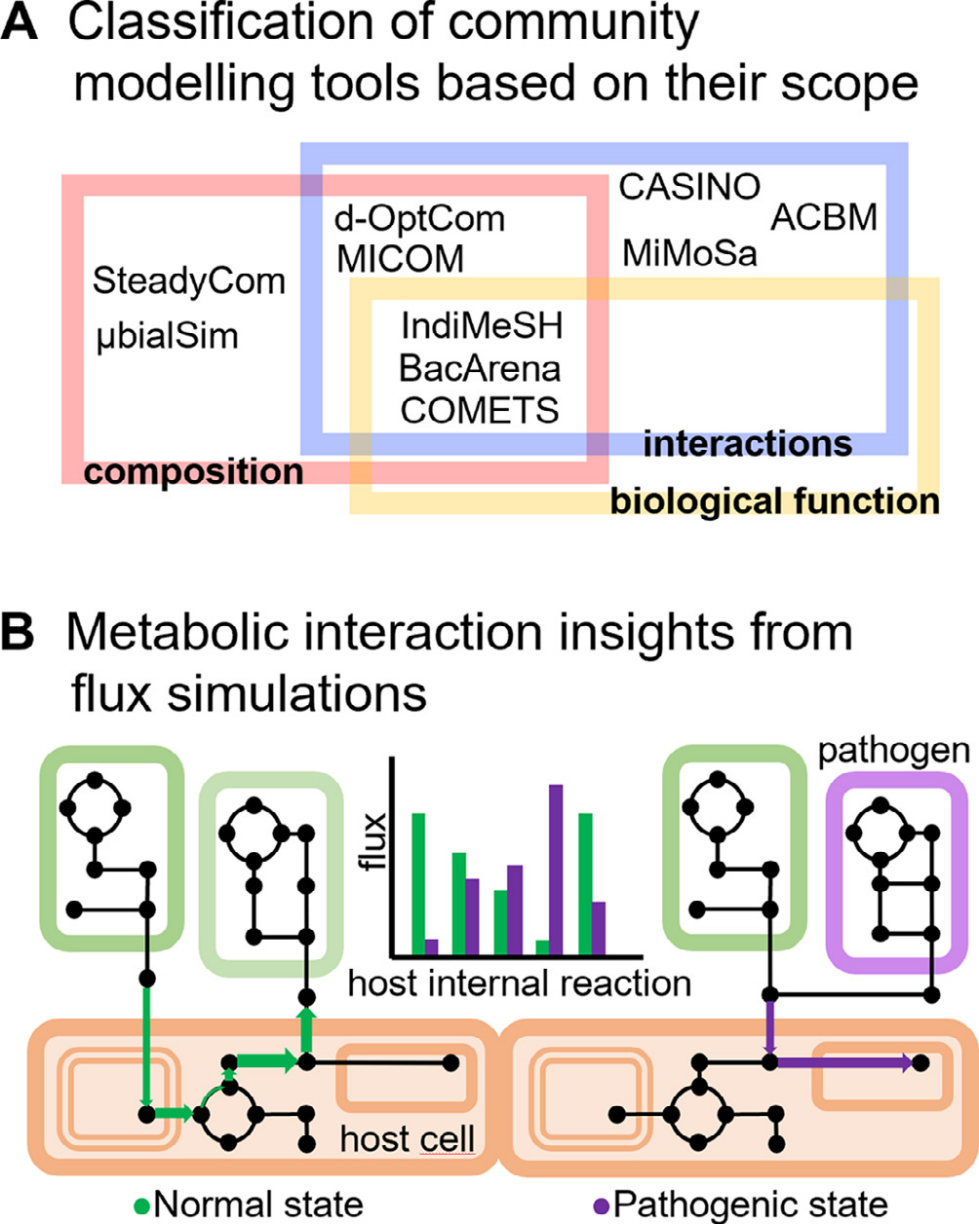
Scope of different metabolic modelling tools for the study of microbial communities. **A** Metabolic modelling tools can be classified based on their capabilities for probing key ecological aspects of microbial communities, namely: composition, interactions and biological function. **B** Rooted on constraint-based methods such as Flux Balance analysis, metabolic models can provide insights into the metabolic state of a host cell (e.g., enterocyte) upon invasion of a pathogen or in the presence of commensal bacteria by comparing the flux distributions in the two conditions.

**Fig. 3:**
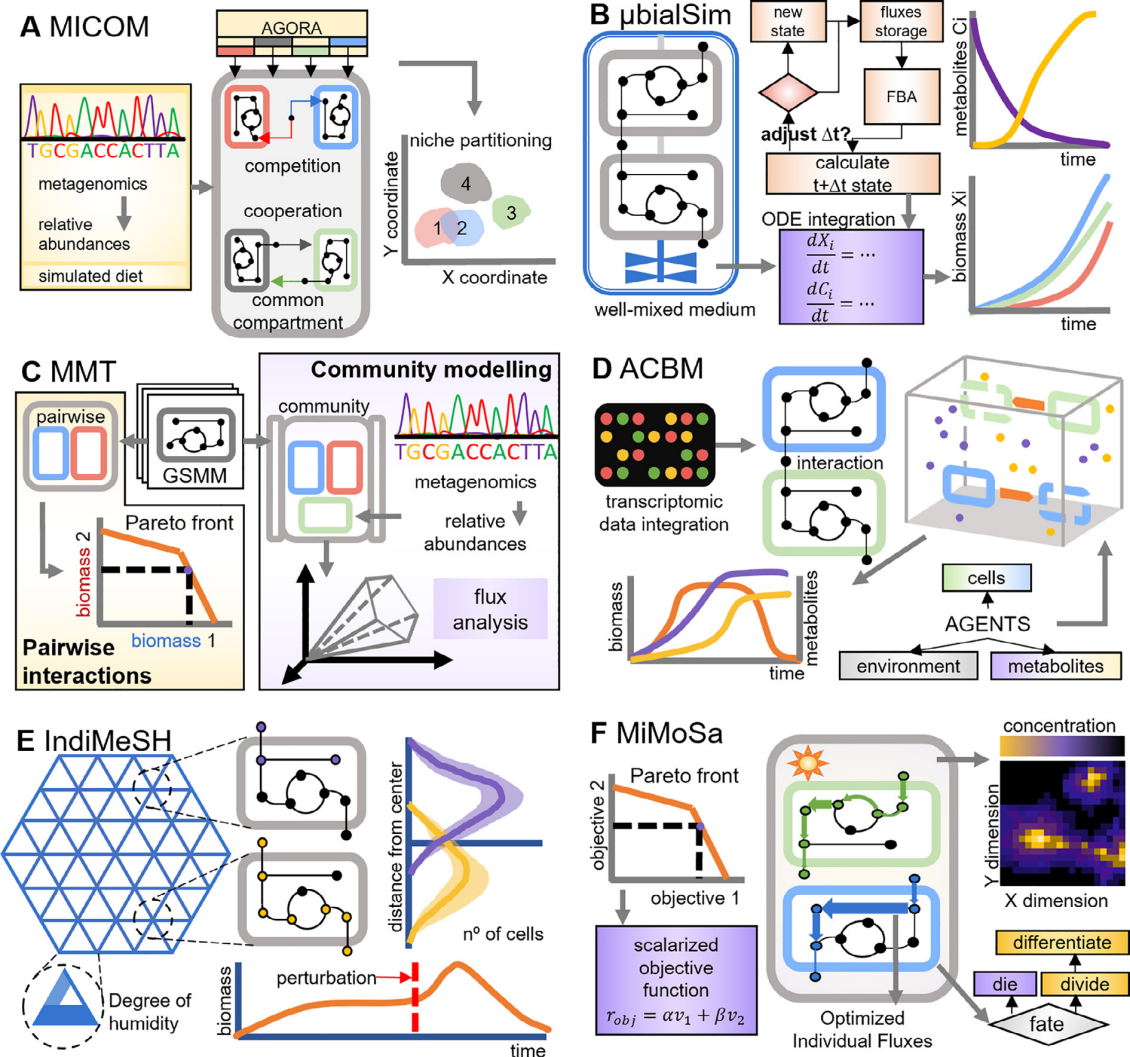
Schematic representations of different genome-scale metabolic modelling tools for probing microbial communities in space and time. **A** MICOM computes microbial interactions in the human gut by integrating abundance data from metagenomic samples and GSMMs obtained from AGORA under defined conditions (e.g., western diet). **B** μbialSim describes the time-course growth and metabolic consumption/production of multiple microbial species in a well-mixed culture. **C** MMT employs a simple steady-state approach for studying pairwise interactions within the community and building the associated Pareto front. Community models generated with MMT differ from others in that non-traditional compartments can be considered. **D** ACBM uses a 3D cubic space (agents) to simulate bacterial chemotactic behavior in a microbial community. This method also enables the incorporation of transcriptomic data using constraint-based methods to characterize different phenotypes within the community. **E** IndiMeSH uses a 2D lattice to simulate the dynamics and movement of bacteria and diffusion of chemical compounds within a pore-network. IndiMeSH can simulate changes in abundance of cells upon external perturbations as well as the differentiation of the microbial population according to the local environment. **F** MiMoSa employs a dynamic multi-objective hybrid constraint-based approach for simulating concentration changes in time and space for different species. Individual optimized fluxes for each cell type (blue and green weighted arrows) are used to update the chemical concentrations of the 2D environment and also to decide whether cells divide or differentiate based on their size. (For interpretation of the references to colour in this figure legend, the reader is referred to the web version of this article.) (For interpretation of the references to colour in this figure legend, the reader is referred to the web version of this article.)

Genome-scale metabolic tools can be categorized according to their mathematical features or biological/ecological scope. Using the latter criterion, we review the most relevant and recent tools for modelling the composition, interaction and biological function of microbial communities (Fig. 2A). The presented tools are mostly based on CBMs, and as such, they provide mechanistic insights into the metabolic interactions for example between microbial species and their impact on the (human) host (Fig. 2B). The main features of the reviewed tools are illustrated in Fig. 3.

### 3.1 Prediction of composition in microbial communities

Metagenome samples can be sequenced for their 16S rRNA content to determine the species and their abundance – as determined by the number of reads – in the microbial community [52], [53]. The reads abundance data can then be integrated into metabolic models for evaluation of the microbial community behavior. Using this strategy, a customizable collection of metabolic models (collectively called MICOM, Fig. 3A) [35] was applied to study the microbial gut composition of Swedish and Danish individuals (healthy or with diabetes) consuming a Western Diet [54]. In the study, 186 metagenome samples were gathered and relative abundance data were used to construct a community model and simulate its behavior (compartment per guild approach). The metagenomic data was relevant for the differential selection of bacterial genera, and the community objective involves a trade-off between the community and individual growth.

A recent CBM for microbial composition prediction is SteadyCom [46]. SteadyCom seeks to determine the optimal microbial composition that supports the maximum community growth rate at steady-state. In particular, SteadyCom predicted the dominance of the bacterial phyla Bacteroides and Firmicutes in the gut microbiota [46]. Notably, the method formulates and solves a finite series of linear optimization problems that do not depend on the number of species considered in the community. A limitation of this approach is that it assumes the same specific growth rate for all members of the community. This assumption is known to hold over periods of time [42]; however, it cannot be used to model dynamic conditions of microbial growth and communities.

The change of microbial composition over time is of great importance for understanding biological interactions. For this task, d-FBA is the most used CBM for metabolic modelling. μbialSim is a d-FBA-based simulator that describes the dynamic evolution of the biomass composition and metabolite concentrations in a microbial community growing in batch culture (Fig. 3B) [49]. Notably, μbialSim incorporates a numerically robust integration scheme that enables efficient simulation of a large number of species avoiding infeasible trajectories, e.g., reaching negative concentrations. The application of this method enabled satisfactory simulation of a 773-species human gut microbiome that exhibited a complex and dynamic pattern of metabolite exchanges. μbialSim is, however, limited to well-mixed environments, which could miss critical metabolic phenomena arising from spatial interactions, especially when it comes to simulation of the gut microbiota.

Simulation of community growth in time and space and assuming different objectives has also been achieved. For instance, d-OptCom [48], an extension of OptCom [47], uses a multi-level multi-objective optimization approach maximizing the overall community biomass and individual specific growth rates at each time step, which ultimately yields the time-course profile of the consortium composition. As μbialSim, d-OptCom assumes perfect mixing in the community. In contrast, COMETS [22] uses d-FBA to simulate metabolite production/consumption by different species, and diffusion on a discretized lattice to simulate spatial and temporal gradients. Similarly, BacArena [55] has also described the evolution and spatial distribution of different species and metabolites. However, it employs a hybrid agent-based framework that combines FBA and Michaelis-Menten kinetics for describing the dynamics of the agents. Notably, this approach enabled predicting not only the abundances but also the spatial distribution of members of a representative consortium and niche separation in the human gut [55].

### 3.2 Probing metabolic interactions within microbial communities

Pairwise ecological interactions can be broadly considered as detrimental, beneficial, or neutral. Many of these interactions can be captured by GSMMs. In a metabolic modelling context, competition involves the uptake of common substrates (detrimental) from the environment, whereas cross-feeding (syntrophy) describe the exchange of metabolites from one organism to another (beneficial). The previously mentioned MICOM method has been applied to simulate cross-feeding interactions involved in SCFA production [35] (Fig. 3A). Computational results from MICOM were unusual, as they pointed to *Bacteroides* and *Eubacterium* genera to be responsible for acetate and propionate consumption, respectively, while *Shigella* and *Escherichia* genera were responsible for their production, which is not been commonly reported *in vivo*. Additionally, MICOM showed that competition was a common interaction between almost all members of the gut microbiota due to the large set of metabolites that could be consumed. Notably, simulation results also suggested niche partitioning in the gut microbiota (see [56], [57] for other examples); there is either consumption of fibers and starches (e.g., inulin, xylan, and pectin) by a group, or consumption of Branched Chain Amino Acids (BCAAs) by another.

By using different metabolic reconstructions from various sources, the Microbiome Modelling Toolbox (MMT) [58] enables studying microbial interactions within a community in a specific context (e.g., diet, fecal compartments) using relative microbial abundances as input (Fig. 3C). Following the construction of a community model contextualized with experimental data [59], this tool indicated a metabolic cross-talk between *Lactobacillus rhamnosus* and Caco-2 cells. CASINO [21] is another tool that enables similar analyses. By defining a certain diet and microbial abundance (e.g., estimated from 16S rRNA sequencing), CASINO enabled simulation of different metabolite production and consumption profile with the added benefit of predicting the individual contributions of each species, e.g., SCFAs production. The application of this tool is, however, limited to small communities. d-OptCom, already mentioned above, has also been applied for simulating cross-feeding metabolic interactions between *E. coli* auxotrophs [48].

As mentioned in the previous section, agent-based modelling methods are both useful for capturing the dynamic and spatial distribution of species and flexible for integrating results derived from FBA. For instance, Agent and Constraint-Based Modelling Framework (ACBM, Fig. 3D) [18] satisfactorily simulated the cross-feeding between *Faecalibacterium prausnitzii* and *Bifidobacterium adolescentis* in the gut microbiota. In this case, *F. prausnitzii* was predicted to produce four times the amount of butyrate in co-culture with *B. adolescentis*, which was in agreement with experimental data [60]. Similarly, IndiMeSH (Fig. 3E) [24] is a hybrid CBM and ABM tool, which predicted trophic relationships between different populations of a bacterial species, namely *Pseudomonas putida* and *Pseudomonas veronii*. More specifically, simulations predicted the growth of facultative anaerobes with the concomitant production of acetate in the center of a soil aggregate under wet conditions, whereas in the outer region, aerobic growth was sustained by the produced acetate. Another agent-based method called MiMoSa [25] uses a similar approach as the previous method, but it employs multi-objective optimization instead (Fig. 3F). This tool was employed to model two different cell types – photoautotrophic and diazotrophic – of *Trichodesmium erythraeum,* and accurately predicted the differential activation of biochemical pathways by varying the weights of the objective function according to cell size. This function prioritized biomass production at small cell sizes and production of either glycogen or cyanophycin for larger cell sizes below the threshold of cell division. Although these tools have been deployed for simulating environmental habitats, they can be readily applied to represent other situations such as proliferation of infectious bacteria in human tissues like the lungs or aerated epithelial tissues colonized with microbes like the mouth [24].

Finally, the study of pairwise interactions of mutualistic nature, or between two closely interrelated cell types, might be complemented with the quantification of the metabolic support that each species brings to the other. To this task, the Metabolic Support Index (MSI) quantifies the number of reactions of a metabolic network that are *enabled* (i.e., increased metabolic capacities) when the metabolic network of another organism interacts in a simulated co-culture [61]. Although MSI is not a method *per se*, it can be readily implemented within a CBM framework, for example, for better understanding how mutualistic interactions may be affected by media composition.

### 3.3 Inferring biological functions under different environmental conditions

The biological role or function of a community depends inevitably on environmental factors such as nutrients and oxygen availability, light intensity, shear forces, water activity, or temperature. To appropriately capture this interdependency, the time evolution and spatial distribution of the cells and molecules need to be described. These are particularly the cases of ACBM, InsiMeSH, and MiMoSa.

IndiMeSH predicted the spatial distribution and metabolic behavior of *Pseudomonas stutzeri* A1501 under wet and dry conditions of soil. Under dry conditions, the entire population was in fully aerobic metabolism thanks to oxygen penetration, whereas under wet conditions, nitrate consumption was predicted (Fig. 3E). Notably, the latter resulted in overflow metabolism and the production of acetate by a denitrifying subpopulation at the center of the lattice [24]. As opposed to IndiMeSH, ACBM models single agents in a 3D cube across time. By incorporating transcriptomic data from *Escherichia coli* MG1655 in the form of constraints for metabolic simulation, this tool predicted the emergence of two distinct phenotypes in different locations determined by the glucose concentration at the end of the log phase (Fig. 3D). One of the drawbacks of this approach is that it assumed no cost for motility and momentaneous starvation, i.e., no maintenance energy requirement considered. In turn, MiMoSa enabled spatial and time-course simulation of how changes in the microenvironment – particularly light intensity – affect the metabolic flux distribution of single cells (Fig. 3F). In the *T. erythraeum* case study, MiMoSa predicted the excretion of 20% of the fixated nitrogen into the environment, improving previous estimates in terms of the energetic costs [62].

In the context of the effect of the gut microbiota on human health, there have been two recent studies that showcased how CBMs can probe biological functions [34], [58]. In one study, MMT was used to generate personalized community models based on metagenomic data simulating an average European diet (Fig. 3C). FBA calculations yielded the maximum potential capability of conjugation and biotransformation of bile acids for each individual model, which is relevant for inflammatory bowel disease patients. Similarly, the metabolic potential was analyzed in microbiomes obtained from Parkinsońs disease patients. A relationship between the potential of maximal production of certain metabolites and constipation – among other traits of Parkinsońs disease – was found, which suggests that alterations in the gut microbiota composition may play a role in metabolic potential and related Parkinson’s disease symptoms.

## 4 Challenges and outlook

GSMMs are excellent tools for exploring the properties of microbial communities as they are suited for the application of a variety of computational methods [23]. From predicting the composition of environmental communities or host-associated consortia (e.g., human gut) [46], to understanding the biological function of individual species in the context of their community and environment [24], [34], [58], there is a growing family of methods that have been developed to resolve these fundamental questions, and to grasp even more complex features related to dynamics and spatial distribution microbial communities. Despite the many virtues of GSMMs and the associated tools, there are still many challenges hindering further progress.

Construction of predictive GSMMs from GENREs remains a laborious and challenging task. Although there are different pipelines and workflows available for streamlining the reconstruction process [26], [27], detailed manual curation is still required for arriving at high-quality networks. For instance, recent comprehensive GENREs resources (e.g., AGORA [33]) contain several hundreds of reconstructions, which are mostly developed in a semi-automated fashion. Careful manual curation and revision are thus needed to ensure the quality of the network. This difficulty is only exacerbated when modelling the gut microbiota, as many of its members lack detailed biochemical information of their metabolism and functional annotation of their genes [63]. Furthermore, even if single-species GSMMs can be satisfactorily constructed, formulation of a community model is not a trivial task as different strategies can be employed. The selection of the most appropriate strategy will mostly depend on the underlying assumptions, research question, and available data, which may largely vary for different communities [45]. For instance, what are the most relevant interactions within the community remains an open question for most relevant microbial systems [64].

A critical limitation of current genome-scale metabolic modelling tools is related to their temporal and spatial resolution capabilities for modelling microbial communities. This is particularly relevant for the human gut microbiota. The gut microbiota is a highly heterogenous group of microbes spatially distributed along various sections of the human intestines. It is known that the adopted spatial organization has an important effect on human health, as it can promote different types of Inflammatory Bowel Diseases (IBDs) [65]. A recent study using a combination of CBMs modelled representative bacteria (5) of the human gut and yielded valuable insights about the role of oxygen as a key contributing factor in the longitudinal and radial spatial organization (composition) of the simulated community [66]. Some of the tools reviewed here display similar capabilities for modelling spatial distributions with even greater detail [18], [22], [25], however, they are all only suitable for describing small communities. Furthermore, in all these cases, there is no consideration as to how the environment – or host in the case of the human gut – will respond. In the latter case, multi-scale models of the human body [67] could be constructed and adapted for capturing the dynamics and interplay between host metabolism, microbiota, and possibly diet. In this way, more holistic therapeutic strategies may be designed for more personalized site-specific perturbations of the gut microbiota.

A more detailed understanding of the composition, metabolic interactions, and ultimately, biological functions of microbial communities within a given environment will enable the design of synthetic consortia with desired functions. Synthetic communities have already been used in bioremediation [68], and there is growing interest in designing consortia with therapeutic activities to treat relevant pathologies [7], [8]. Experimental efforts can benefit from computational methods as they enable narrowing the vast design space to a more practical set of consortia. Framework based on dynamic modelling of GSMMs could be extremely helpful in predicting emergent properties of microbial communities. However, they have not been applied to the design of large consortia due to the combinatorial nature of the problem. To this task, other tools – typically graph-based – have been proposed as they can cope with the massive scale at the cost of yielding only qualitative predictions. For instance, Miscoto [69] has been shown to exhaustively enumerate millions of possible minimal consortia (i.e., with the least number of members) capable of performing a defined metabolic task, i.e., producing a metabolite from a set of substrates. This method is suited for screening large sets of compounds and networks and enables redundancy analysis of metabolic tasks. The identification of keystone species is also critical for properly modelling in microbial communities [19]. These species are essential for the development of the community and, when removed, they drastically disrupt the community and environment. Eventually, experimental studies will need to be conducted to determine the presence/absence of such microbes in the synthetic community [70]. Importantly, by leveraging the power of computational methods, the biological function of keystone species will be identified and preserved – or even engineered using *in silico* tools [71] – in the synthetic consortia.

## CRediT authorship contribution statement

**Álvaro Altamirano:** Conceptualization, Writing - original draft. **Pedro A. Saa:** Conceptualization, Writing - original draft, Writing - review & editing, Funding acquisition. **Daniel Garrido:** Conceptualization, Writing - review & editing, Funding acquisition.

## Declaration of Competing Interest

The authors declare that they have no known competing financial interests or personal relationships that could have appeared to influence the work reported in this paper.
